# Passing Decisions in Football: Introducing an Empirical Approach to Estimating the Effects of Perceptual Information and Associative Knowledge

**DOI:** 10.3389/fpsyg.2018.00361

**Published:** 2018-03-22

**Authors:** Silvan Steiner

**Affiliations:** Institute of Sport Science, University of Bern, Bern, Switzerland

**Keywords:** decision making, logistic regression, pass prediction, position data, team sports

## Abstract

The importance of various information sources in decision-making in interactive team sports is debated. While some highlight the role of the perceptual information provided by the current game context, others point to the role of knowledge-based information that athletes have regarding their team environment. Recently, an integrative perspective considering the simultaneous involvement of both of these information sources in decision-making in interactive team sports has been presented. In a theoretical example concerning passing decisions, the simultaneous involvement of perceptual and knowledge-based information has been illustrated. However, no precast method of determining the contribution of these two information sources empirically has been provided. The aim of this article is to bridge this gap and present a statistical approach to estimating the effects of perceptual information and associative knowledge on passing decisions. To this end, a sample dataset of scenario-based passing decisions is analyzed. This article shows how the effects of perceivable team positionings and athletes' knowledge about their fellow team members on passing decisions can be estimated. Ways of transfering this approach to real-world situations and implications for future research using more representative designs are presented.

## Introduction

Approaches explaining the mechanisms underlying interpersonal coordination in interactive sport teams have been the focus of various scientific contributions (e.g., Cannon-Bowers and Bowers, 2006; Gorman, 2014; Araújo and Bourbousson, 2016; McNeese et al., 2016). One major difference between some of these approaches is the importance they attribute to different information sources for decision-making purposes. Some highlight the role of the perceptual information provided by the current game context (e.g., Araújo et al., 2006; Fajen et al., 2009). Others point to the role of team plans and other knowledge-based (internal) information that athletes have regarding their team environment (e.g., Annett, 1996; Eccles and Tenenbaum, 2004, 2007; Reimer et al., 2006). Still other have called for integrative perspectives that consider both sorts of information (e.g., Nitsch, 2009; Gorman, 2014; see also Pedersen and Cooke, 2006; Duarte et al., 2012; Cooke et al., 2013; McNeese et al., 2016).

Recently, an integrative perspective that considers the simultaneous contributions of multiple information sources to enabling coordination in interactive team sports has been presented (Steiner et al., 2017). The perspective views decision-making from the angle of athletes adapting their own goal-directed behavior to that of other team members in order to enable interpersonally coordinated team behavior in dynamic group-task environments. The environments in open-type sports constantly change when positions of team members, opponents and the ball are altered. Athletes must keep themselves informed about these changes by visually monitoring their environment (Tenenbaum, 2003). The integrative perspective (Steiner et al., 2017) considers different ways in which information picked up by the visual system affects decision-making. The direct impact of perceptual information on behavior (e.g., through the perception of affordances; see Gibson, 1979; Silva et al., 2013) represents the bottom-up part in organizing directed behavior. It is is considered an integral part of coordinated team behavior. At the same time, the framework considers the role of internal knowledge that players associate with their perceptual environment in a cognitive top-down process. In this case, perceived information is passed on for higher level processing. Assumedly, this higher level processing consists of an ongoing interaction between working memory and long-term memory (Tenenbaum, 2003). Depending on situation- and athlete-specific characteristics, various sources of information are assumed to be of different relevance to the decision-making process. The charactersitics may refer to given time constraints, the novelty of a situation, an athlete's perceptual attunement to key features of a situation or other knowledge structures resulting from previous experience with similar situations. In an example involving passing decisions, Steiner et al. (2017) illustrate how perceptual information about the positioning of team members and opponents define task constraints on a moment-to-moment basis and inform athletes about available and unavailable passing opportunities (see also Araújo et al., 2006; Fajen et al., 2009; Silva et al., 2013). If time allows, athletes may complement the perceptual information about the positioning of team members with knowledge they associate with their team members (e.g., Annett, 1996; Eccles and Tenenbaum, 2004, 2007; Nitsch, 2004; Rentsch and Davenport, 2006; Rico et al., 2008; Tenenbaum and Land, 2009; Seiler, 2014). If a ball carrier perceives two team members standing equally open for a shooting attempt, then her associative knowledge about the higher task-specific strenghts of one of them could be the decisive factor to pass the ball to that team member (Johnson, 2006). While Steiner et al. (2017) illustrate the theoretical simultaneous involvement of perceptual and knowledge-based information in decision-making, they do not provide a precast way of determining the contributions of various information sources empirically.

The aim of this article is to bridge this gap and present an approach to estimating the effects of perceptual information and associative knowledge on passing decisions. This approach will be shown using scenario-based data. Ways of transfering the approach to real-world situations and the implications for future research using more representative designs will be presented.

### Sources of perceptual information

Information provided by situational game contexts is essential for teams in coordinating their behavior in real time (e.g., Eccles and Tenenbaum, 2004, 2007; Araújo et al., 2006; Silva et al., 2013; Travassos et al., 2013; Gorman, 2014; McNeese et al., 2016). With regard to passes, the current positions of team members are relevant perceptual information because they constrain passing opportunities to nearby areas (Gorman, 2014; Vercruyssen et al., 2016). With ten pass receivers potentially available in football, multiple passes to various team members may be functional in terms of team performance (Steiner et al., 2017; see also Oesterreich, 1981). Presumably, athletes consider perceptual information in deciding which pass to make. Providing a theoretical example of a temporary passing affordance, Fajen et al. (2009) describe how an unblocked passing path to a teammate at a given moment in a match presents an opportunity to pass to that teammate. Various studies have reported relationships between the distance of defenders to ball trajectories (e.g., shooting or passing paths) and the frequency of ball interceptions, corroborating this perceptual information's relevance to passing decisions (e.g., Travassos et al., 2012; Vilar et al., 2013). Team members' positions relative to the ball carrier may represent further perceptual information used in passing decisions. Given the primary goal of scoring more points than the opposing team, there is a certain need to pass the ball forward to take it into scoring positions near the goal (e.g., Carling et al., 2015). Whether a team member is closer to the opponents' goal than the ball carrier or further away from it represents perceptual information that discloses the goal-approximative consequences to be expected after a corresponding pass (Oesterreich, 1981). At the same time, passes must have a chance of reaching the intended receiver and lie within each agents' range of capabilities (Fajen et al., 2009; Vercruyssen et al., 2016; see also Bandura, 1997; Tenenbaum, 2003; Nitsch, 2004, 2009). It has been shown that passes to distant team members are made less frequently than passes to nearby team members (e.g., Rampinini et al., 2009; Hjelm, 2011). While long passes may create new opportunities for the team (and be worth a try), they generally have a higher risk of being off-target and missing the intended receiver. Team members' large distances from the ball carrier is perceptual information that could prevent these team members from being perceived as viable passing opportunities. Finally, when opponents defend team members tightly, they jeopardize the success of a pass because it may be intercepted (Johnson, 2006; Hjelm, 2011; Vilar et al., 2014; Macquet and Kragba, 2015). How tightly team members are defended represents further perceptual information athletes must consider.

### Athletes' knowledge about their team members

In addition to perceptual information, the role of conceptually-driven cognitive processes in recognizing decision-relevant factors in game situations has been discussed, the main point being that the encoding of situational information is mediated by the perceiver's knowledge of what he is observing (Willams et al., 2006). This mediating role of cognitively represented knowledge has been discussed in terms of its various contributions to sports (e.g., Eccles and Tenenbaum, 2004; Tenenbaum and Lidor, 2005; Reimer et al., 2006; Rentsch and Davenport, 2006; Nitsch, 2009; Tenenbaum and Land, 2009; Vilar et al., 2012). Generally, it is argued that this approach enables experts to see beyond the perceptual information itself (Gobet, 1998; Willams et al., 2006).

The knowledge athletes have regarding their fellow team members has been argued to be of special relevance (e.g., Annett, 1996; Tenenbaum, 2003; Eccles and Tenenbaum, 2004, 2007; Johnson, 2006; Reimer et al., 2006; Rentsch and Davenport, 2006; Eccles, 2010; Seiler, 2014; for contributions unrelated to sports, see also Mathieu et al., 2000; Cannon-Bowers and Salas, 2001; Langan-Fox et al., 2004). Annett (1996) uses the term “cognitive people models” to refer to the knowledge and understanding of fellow players, such as knowledge of their strengths and weaknesses (see also Eccles and Tenenbaum, 2004). Such cognitive models of team members can be important in many game situations, both offensive and defensive ones. In Steiner et al.'s (2017) example, they contribute, in combination with perceptual information, to the ball carrier's perspective on the degree to which specific team members represent viable opportunities for passes.

In the following, a statistical method of estimating the effects of four kinds of perceptual information and athletes' cognitive models of their team members on passing decisions will be shown. Based on the above considerations, five hypotheses are formulated and tested to illustrate the statistical approaches using an applied example. It is hypothesized that the probability of deciding to pass to certain team members increases when passing lanes to team members become more open (Hypothesis 1), when they stand closer to the opponent's goal than the ball carrier (Hypothesis 2), when they are within close reach of the ball carrier (Hypothesis 3), and when they are loosely defended (Hypothesis 4). Cognitive people models (Annett, 1996) represent a class of internal information sources. They are included to illustrate the possibilitiy of estimating the effects of both perceptual and knowledge-based information sources on passing decisions simultaneously. It is hypothesized that team members subjectively represented as having high-level football-related skills have a higher probability of being passed the ball than team members represented as having low-level skills (Hypothesis 5). No hypothesis considering the relative effects of the various information sources on passing decisions is formulated.

## Materials and methods

### Subjects

The complete athletic staff of two football teams playing in the fourth league of the Swiss Football Federation were invited to provide answers for the sample dataset. Out of the 46 players contacted, 30 responded to the first part of the survey (3 wing-backs, 7 central defenders, 13 midfielders, 3 forwards and 4 midfielders/forwards; *M* = 23.4 years, *SD* = 4.5). On average, the athletes had been playing football for 16.3 years (*SD* = 5.4) and had been part of their current teams for 9.7 years (*SD* = 6.2). Seventeen players also completed the second part of the survey (1 wing-back, 4 central defenders, 7 midfielders, 3 forwards, 2 midfielders/forwards). On average, they were significantly older and had been playing football for longer than the players who dropped out [*t*_(45)_ = 3.64, *p* < 0.01 and *t*_(45)_ = 2.58, *p* < 0.05, respectively]. The two groups did not differ with regard to the number of years of membership in their current teams [*t*_(45)_ = 0.73, *p* = 0.47]. The data were collected at the end of the season, which included a total of 22 league matches. During the season, both teams practiced three times a week. Before the athletes were contacted to ask for their consent to participate in the study, the two team coaches were asked for their general permission to collect data regarding their athletes. The study was conduct in accordance with the protocols Nr. 2016-10-000003 and Nr. 2017-02-00001 approved by the Ethics commission of the Faculty of Human Sciences of the University of Bern. All subjects gave written informed consent in accordance with the Declaration of Helsinki.

### Procedure

**Figure 1:**
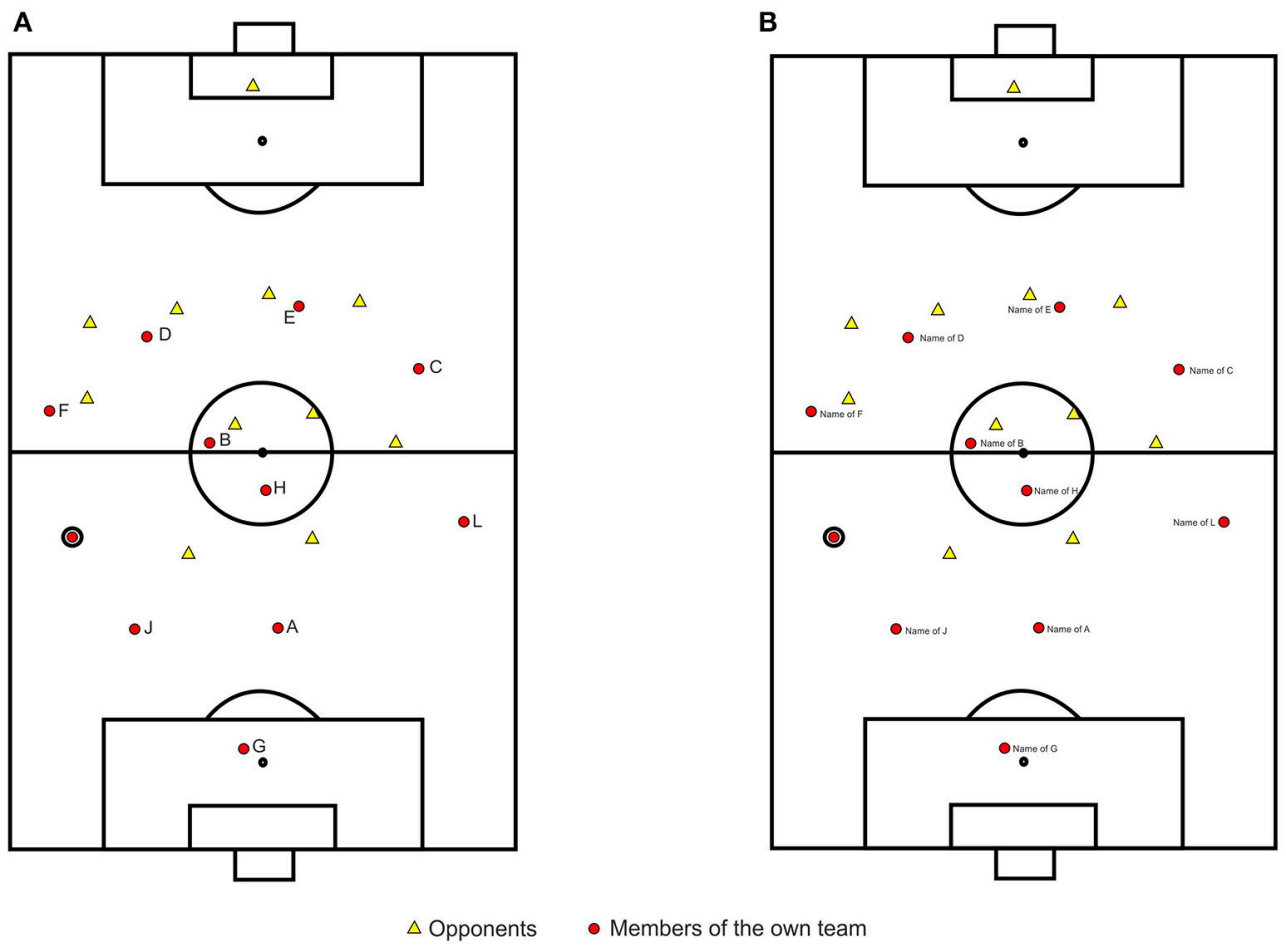
Game scenario as used in the study during the anonymous condition **(A)** and the condition with name labels **(B)**. The encircled player represents the ball carrier.

In personal meetings, the coaches provided information about the game strategy they followed, the lineups of their teams, and the individual athletes on those teams. This information was used to graphically illustrate 40 game situations that were customized with respect to each of the two teams. The game situations were created in CorelDRAW (Version X7). All game situations showed the participating teams having ball possession. Each of the ten field players was the ball carrier in four out of the 40 scenarios. The scenarios were presented to the coaches to determine whether the scenarios were representative examples of their teams' play. If required, the situations were adapted according to the provided feedback. Figure 1 shows one of the game scenarios used in the study.

Data were collected via two Internet-based surveys (limesurvey.org). Invitations to the second section were sent 4 weeks after invitations to the first section. In the first survey, the 40 team-customized game scenarios were presented to the athletes in randomized order. The game scenarios were programmed to appear in the center of the display at fixed aspect ratio and adapted to the actual screen height. The survey was not available on mobile devices (e.g., tablets or smart phones). Participants were asked to assume the perspective of the encircled player with the ball and state to whom they would pass the ball. During this first phase of data collection, the graphed players remained anonymous and were labeled using alphabetic characters (Figure 1A). The players could indicate their passing decisions by clicking on the corresponding character in a list presented on the left side of the game scenarios. A comment box at the end of the survey was provided. During the first part of the survey, four players used the comment box to state that they would not have passed the ball to any team member in two of the shown situations. Instead, they would have dribbled the ball toward the goal to try to shoot at the goal. In both scenarios the ball carrier was a forward in close position to the opponents' goal with only one defender and the goal keeper between himself and the goal. A team member was positioned slightly behind the ball carrier. Twelf and 20 missing answers to these two scenarios indicated that further players chose not to pass the ball to any team member in the same situations. Obviously, the game scenarios did not present situations in which a pass would be a viable option for continuing the play. The game scenarios were no longer considered in the second phase of data collection and excluded from the analyses. For the second phase of data collection, the remaining 38 scenarios were re-shown to the players. The scenarios were identical to those of the first part except that the names of the team members were indicated in the scenarios (Figure 1B). Participants were then able to use stored information regarding their team members when making passing decisions. After the participants had stated their passing decisions in the second survey, they rated all team members in terms of five items. The items addressed global competencies relevant to football. They referred to technical skills, understanding of the game, game intelligence and ball control (e.g., Williams and Reilly, 2000; Wein, 2004; Schreiner, 2009; Memmert, 2010). The fifth item addressed the team members' overall football capabilities. Table 1 shows the wording of each of the five items. Likert scales from 1 (I don't agree at all) to 9 (I completely agree) were used.

**Table 1:** Wording of the indicator items used for the latent variable people model.

**Item**	
1	{Name of team member} is technically adept.
2	{Name of team member} reads the game well.
3	{Name of team member} is game intelligent.
4	{Name of team member} is good on the ball.
5	{Name of team member} is one of the strongest players on our team.

### Data preparation

**Figure 2:**
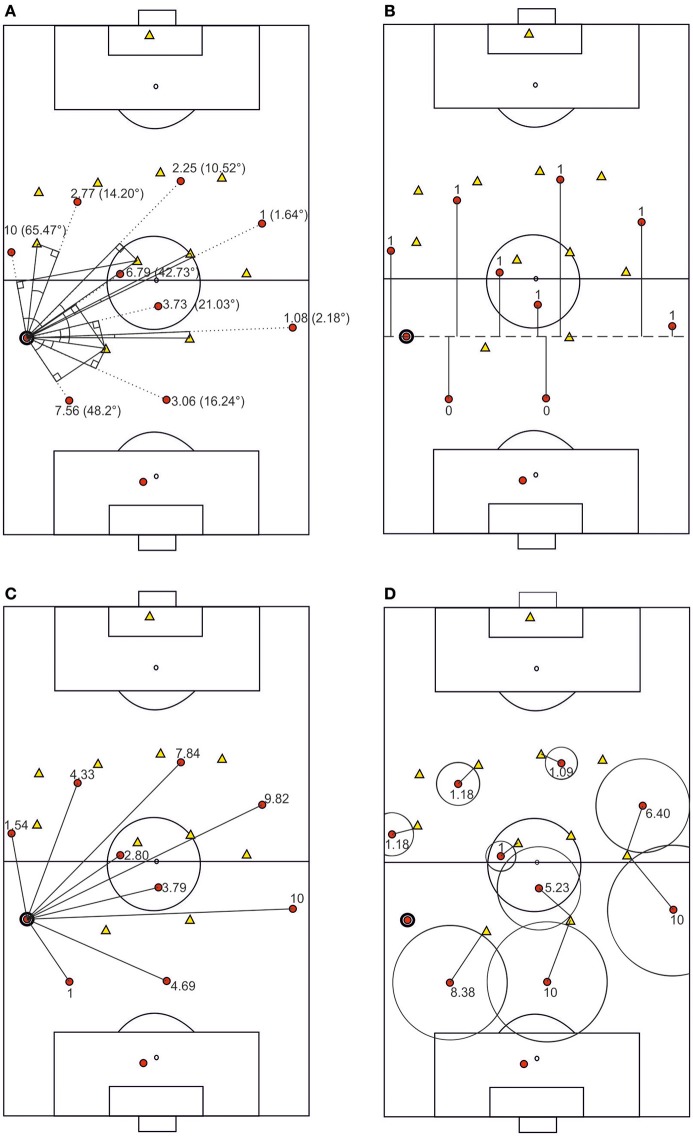
Visual illustration of the four variables: openness of passing lane **(A)** position relative to the ball **(B)** distance **(C)** and defensive coverage **(D)**. The numbers represent normalized values, as used in the logistic regression models.

The data were arranged in a long data file (Heck et al., 2014). For each passing decision made by a participant, 11 cases were added to the dataset. Each case represented one of the 11 team members within a given scenario. A variable was used to code the ball carrier (−1), the pass receiver (1), and the non-receivers (0). Variables describing the team members' positions relative to the ball carrier were measured via CorelDRAW's dimensioning tool. These variables represent the perceptual information that is available from the perspective of the ball carrier. The first variable, representing the openness of the passing lane to each team member, was operationalized as the angle between the passing lane (the straight between the ball carrier and a team member) and the straight to the opponent standing closest to this passing lane. The distance of an opponent to the passing lane was measured by drawing the perpendicular to the passing lane through this opponent. The variable is visualized in Figure 2A^1^. The second variable was the team members' relative closeness to the opponents' goal. Team members positioned behind the ball carrier were coded as 0, while those positioned in front of him (closer to the opponents' goal) were coded as 1 (Figure 2B). For the third variable, the Euclidean distances between the team members and the ball carrier were measured (Figure 2C). Finally, the defensive coverage of team members by opposing players was measured. This fourth variable was operationalized as the shortest distance between any opponent and each team member (Figure 2D). Within each of the 38 game scenarios used in the study, the values for the openness of passing lanes, Euclidean distances, and defensive coverage were linearly transformed to values between 1 and 10. In addition to adjusting model sensitivity (e.g., Pina et al., 2017), this transformation helps maintain information about the situation-specific distribution of the predictor variables within the variable values. The predictor variables were z-standardized and screened for univariate outliers. No standardized case outside the absolute value of 3.29 (two-tailed) was found (Tabachnick and Fidell, 2014). Squared Mahalanobis distances indicated the absence of multivariate outliers.

### Statistical analyses

For each situation, the data row of the case representing the ball carrier was excluded from the dataset (e.g., the ball carrier does not represent a passing option to himself). Due to their unique function, goalkeepers were considered qualitative outliers and also excluded from the analyses. Thus, during the statistical analyses, each game situation *i* (*i* = 1–38) answered by a participant *j* (*j* = 1–30) included nine outfield players. These outfield players were described by their values for each of the four variables representing perceptual information sources. Furthermore, they were given the values that participant *j* had assigned them when rating their football-related competencies in terms of five items. Direct binary logistic regressions were calculated to test the effects of the four perceptual information sources on passing decisions. The team members' field positions relative to the ball carrier (in front of him vs. behind him) were introduced as a categorical predictor variable. The openness of passing lanes, team members' distances from the ball carrier, and defensive coverage were included as continuous predictor variables. The data from the anonymous game scenarios were used to test Hypotheses 1–4.

The data from the name-labeled game scenarios were used to test hypothesis 5. In a direct logistic regression, a latent continuous predictor variable named *people model* was included along with the other four predictors. The five items on which the participants rated their team members' football skills were used as indicator variables for the latent predictor variable. When analyzing the effect of the people models, the models relied on each participant's own ratings of his team members (and not an average rating of the team members across all participating players of a team). For a better comparison of their effect estimates with those of the variables representing perceptual information sources, the factor scores were also linearly transformed to values between 1 and 10. Thus, for every participant, the team members with the lowest and the highest factor scores in a scenario were always assigned values of 1 and 10, respectively. The statistical analyses to test Hypothesis 5 were performed in Mplus (Version 7.3; Muthén and Muthén). To compare the data fit of the models with and without the people model predictor, a −2 Log likelihood difference test for nested models was performed (Weiber and Mühlhaus, 2010). The test corresponds to a stepwise (or hierarchical) regression in which all variables representing perceptual information are forced into the model in a first block and the people model is introduced as predictor variable in a second block (Tabachnick and Fidell, 2014). For all logistic regressions, the binomial logit link function was used.

## Results and interpretation

Less than three percent of the game scenarios were excluded from the analyses because participants did not indicate any passing decision or because they did not provide ratings of their team members. In total, 1,740 passing decisions could be analyzed. The lack of inordinately large parameter estimates or standard errors in the regression models provided no reason to suspect a problem with outcome groups being perfectly predicted by any variable or there being too many empty cells (Tabachnick and Fidell, 2014). The converging solutions and absence of exceedingly large standard errors for parameter estimates indicated the absence of multicollinearity. This was confirmed by the fact that no tolerance values <0.1 (Menard, 1995) or VIF values >10 (Myers, 1990) were found.

The Box-Tidwell approach (Hosmer and Lemeshow, 2000) revealed significant interactions between all three continuous predictor variables passing lane, distance, defensive coverage and their corresponding natural logarithms (Wald = 8.72, *p* < 0.05, Wald = 29.56, *p* < 0.001 and Wald = 10.95, *p* < 0.001, respectively). This indicated that there was no linear relationship between these variables and the logit transform of the passing decisions. Predictors with no linearity in the logit can be excluded as continous predictor variables in logistic regression analysis (Tabachnick and Fidell, 2014). As a first step, the variables were recoded into variables with five values. The cutoffs were chosen so that each value included an equal number of cases (the cutoffs for passing lane were 1.47, 2.58, 4.46, 6.86, those for distance were 1.63, 3.02, 4.41, 6.29, and those for defensive coverage were 1.13, 1.72, 2.63, 3.45). This quintile transformation of the predictor variables resulted in a linear relationship between the variables distance and defensive coverage and the logit transform of the passing decisions. This recoding did not, however, result in a linear relationship between the passing lane variable and the logit transform of the passing decisions. In consequence, passing lane was treated as a categorical predictor. The indicator contrast method (SPSS 23) was chosen to compare the various categories of this predictor variable. Category 1 (most tightly defended passing lanes) served as the reference category. Table 2 shows the estimates for the model.

**Table 2:** Coefficient estimates for the variables representing perceptual information regarding passing decisions in the anonymous football scenarios.

**Predictor variables**	**β**	***SE***	**Wald**	***df***	**Exp(*B*)**	**CI95%**
Passing lane			41.256^***^	4		
Passing lane (1 vs. 2)	−0.245	0.127	3.744	1	0.783	0.610; 1.003
Passing lane (1 vs. 3)	−0.180	0.124	2.111	1	0.836	0.656; 1.065
Passing lane (1 vs. 4)	0.131	0.124	1.107	1	1.139	0.893; 1.453
Passing lane (1 vs. 5)	0.405	0.127	10.117^***^	1	1.500	1.168; 1.925
Position to ball line	1.183	0.083	203.017^***^	1	3.263	2.773; 3.840
Distance	−0.320	0.033	96.266^***^	1	0.726	0.682; 0.774
Defensive coverage	0.213	0.026	67.165^***^	1	1.237	1.176; 1.302
Constant	−2.689	0.123	475.026^***^	1	0.068	

*Exp(B), Odds Ratio; CI95%, lower and upper 95% confidence intervals for the odds ratio*.

*** *p < 0.001*.

A sample of *N* = 1140 passing decisions (30 players^*^38 game scenarios) from the first part of the survey were analyzed to test the effects of the variables representing perceptual information on passing decisions. All four variables have significant effects on passing decisions. The significant effect on the part of the passing lane variable indicates that the odds ratio (indicated as Exp(*B*) in Tables 2, 3) for passes differs depending on how open the passing lane to a team member is. Team members with the most open passing lanes (category 5) had 1.5 times higher odds for passes than those with the most tightly defended passing lanes (category 1). Using category 5 as the reference category showed that team members with passing lanes of category 5 had significantly higher odds ratios than team members with passing lanes of all other categories. The odds ratios represent estimates of how differences in a specific kind of perceptual information relates to athletes' decisions to pass the ball. The results support Hypothesis 1. There is a higher probability of passes to team members with open passing lanes.

**Table 3:** Mean reciprocal rank (MRR) and percentages of recall among the top-one, top-two, top-three, and lowest three passing options as indicators of the regression models' pass prediction performance.

**Scenario condition**	**MRR**	**top-1 (%)**	**top-2 (%)**	**top-3 (%)**	**lowest-3 (%)**
Anonymous	0.40	20	33	50	7
With name labels^a^	0.45	22	40	57	7
With name labels^b^	0.46	23	41	56	6

a *Pass predictions based on the regression model without the people model variable*.

b *Pass predictions based on the regression model with the people model variable included*.

The second hypothesis assumed that team members located in front of the ball carrier would have a higher probability of receiving passes. The significance estimate is in line with this hypothesis. Team members positioned in front of the ball have 3.26 times greater odds for passes than those behind the ball.

The third hypothesis assumed that team members in relative proximity to the ball carrier would have an increased probability of being passed the ball. Congruent with this hypothesis, the odds for passes decrease the further away team members are located from the ball carrier. With a change of a team member into a more distant quintile, the odds for a pass decrease by 1/0.726–1 = 37.7%.

The fourth hypothesis assumed that loosely defended team members would have a higher probability of being passed the ball than those who were defended tightly by opponents. In line with this hypothesis, the odds for passes to team members increase the more loosely these team members are defended. When controlling for all other variables, each change in defensive coverage into the looser defended quintile changes the odds for passes by a factor of 1.24. Based on the odds ratios and considering the value range of each predictor variable, differences in how far away team members are located from the ball carrier represent the perceptual information source with the strongest relationship to passing decisions. Compared to the most distant players (5), those closest to the ball carrier had a 3.59 times higher odds ratio for passes [OR(3.59) = 1/exp(0.726^*^(5-1))] (Rudolf and Müller, 2012)^2^. Whether players were positioned closer to the opponents' goal or further away from it than the ball carrier was the second most important type of information: being in front of the ball carrier increased the odds for passes by a factor of 3.26. Compared to the best-defended players (1), those being defended most loosely (5) had a 2.34-fold higher odds ratio for passes [OR (2.34) = exp(1.24^*^(5-1)]. Finally, the smallest effect is found for the openness of passing lanes with a 1.5-fold higher odds ratio for team members with the most open passing lanes (5) as compared to those with the best-defended passing lanes (1).

There are several ways of reporting how strongly this specific set of variables relates to passing decisions. The χ^2^ statistic from the −2 log likelihood test indicates whether the regression model fits the data better than the null model, which includes only the intercept. The significant value shows that this is the case in the sample dataset (χ^2^ = 419.460, *df* = 7, *p* < 0.001). Pseudo *R*^2^ values are another way of reporting a regression model's performance. In this example, the model yields a Cox and Snell *R*^2^ of *CS* = 0.041 and a Nagelkerke's *R*^2^ of *NK* = 0.084. Pseudo *R*^2^-values are difficult to interpret independently (Long, 1997). What constitutes a “good” pseudo *R*^2^-value varies between areas of application (Eid et al., 2011). These measures will be useful in comparing the pass prediction of models including different sets of predictor variables. Another indicator to use in estimating the appropriateness of a logistic regression model is the number of correctly predicted cases. Adopting a procedure used by Vercruyssen et al. (2016), the team members' estimated probabilities of receiving a pass were rank-ordered separately for each scenario and participant. The mean reciprocal rank measure (MRR) was calculated as an indicator of the model's performance in predicting passing decisions:

$$\begin{array}{l} MRR=\frac{1}{n}\overset{n}{\underset{i=1}{\sum }}\frac{1}{rank_{i}} \end{array}$$

In the formula, *n* is the total number of passing situations, and *rank* is the rank of the team member that was passed the ball in a given passing situation. Rank could be a value between 1 and 9. The higher the MRR is, the better the pass prediction. Recall in the top-one, top-two, and top-three consists of the percentage of times the pass receiver was ranked accordingly. Recall in the lowest three is an example measure that indicates the percentage of passes that were predicted rather badly by the four variables. The measures are reported in the upper row of Table 3.

The 17 players who responded to the second part of the survey provided a total of 634 passing decisions (17 players^*^38 game scenarios, 12 missings). One player with four missing passing decisions did not rate his team members. Because these ratings were required for testing the effects of subjective people models on passing decisions, his 34 passing decisions could not be used for the analyses. A final sample of *N* = 600 passing decisions remained. The five indicator items all loaded significantly (*p* < 0.001) on the factor people model. Loadings from λ = 0.81 to 0.92 showed that the items were adequate indicators of the latent construct (Comrey and Lee, 1992). The factors explained 82% of the variance in the five items. A linear relationship between the factor scores and the logit transform of the passing decisions was found (Wald = 1.25; *p* = 0.26). Thus, the factor scores were introduced as a continuous predictor variable to complement the four predictors in the previous regression model. The model fit the data significantly better than the null model (χ^2^ = 286.03 *df* = 8, *p* < 0.001). The −2 log likelihood difference test comparing the regression model without and with the people model variable showed that including the people models significantly improved the model's fit to the data (χ^2^ = 7.069, *df* = 1, *p* < 0.01). The results of the model with the people model variable are shown in Table 4. In addition to the effects of the perceptual information sources, the model yields a significant effect on the part of people models on passing decisions. When a team member's score in the people model construct increases by one unit, his odds for receiving a pass increase by a factor of 1.05. Pseudo *R*^2^ measures indicate that *CS* = 0.052 and *NK* = 0.104. Measures of the pass prediction performance of a model without and a model with the people model variable are shown in Table 3 (middle and lower row, respectively). Overall, the findings support Hypothesis 5: athletes more often chose to pass the ball to team members that they had subjectively represented as having higher-level football-related skills as compared to those they had represented as having lower-level skills.

**Table 4:** Coefficient estimates for the variables representing perceptual information regarding passing decisions in the football scenarios with name labels.

**Predictor variables**	**β**	***SE***	**Wald**	***df***	**Exp(*B*)**	**CI95%**
Passing lane			16.485^**^	4		
Passing lane (1 vs. 2)	−0.162	0.170	0.913	1	0.850	0.609; 1.186
Passing lane (1 vs. 3)	−0.123	0.168	0.539	1	0.884	0.637;1.228
Passing lane (1 vs. 4)	−0.028	0.172	0.027	1	0.972	0.694; 1.361
Passing lane (1 vs. 5)	0.347	0.173	4.016^*^	1	1.415	1.008; 1.988
Position to ball line	1.304	0.115	128.168^***^	1	3.682	2.938; 4.615
Distance	−0.394	0.045	78.014^***^	1	0.647	0.618; 0.736
Defensive coverage	0.278	0.038	54.832^***^	1	1.321	1.227; 1.422
People model	0.046	0.017	7.048^**^		1.047	1.012; 1.083
Constant	−2.979	0.210	200.522^***^	1	0.051	

*Exp(B), Odds Ratio; CI95%, lower and upper 95% confidence intervals for the odds ratio*.

* *p < 0.05*,

** p < 0.01, and

*** *p < 0.001*.

## Discussion

The presented approach using exemplary, scenario-based passing decisions illustrates a means of estimating the effects of various information sources on passing decisions in football. It provides a potential research template to complement and test Steiner et al.'s (2017) theoretical assumptions about the involvement of various information sources in the decision-making processes underlying interpersonal coordination. The estimated effects of the variables representing perceptual information reveal how specific information relates to passing decisions and thus indicate that information's relevance to the decisions. The significant effects are based on recurring patterns of situational team alignments and the passing decisions made in such game situations. They indicate a certain consistency in the athletes' use of perceptual information across the various scenarios to determine where to pass the ball. In the exemplary dataset, open passing lanes, being positioned in front of the ball carrier, relative proximity to the ball carrier, and loose defense on the part of opponents were all factors that increased the chance of passes to given team members.

The article illustrates how the effects of internal information sources (associative knowledge) on passing decisions can be tested at the same time as the effects of external (e.g., perceptual) information sources. Players' subjective models of their fellow team members are one example of associative knowledge. The results of the sample dataset indicate these models' potential relevance to decision making. In the game scenarios used, players showed a tendency toward passing the ball to team members they had represented as athletes with high-level football-related skills. The article shows how this tendency can be compared to the effects various perceptual information had on passing decisions. For example, based on the estimated odds ratios, the effect of people models on passing decisions is rather small in comparison to the effects of distance and defensive coverage. Finally, the four variables representing perceptual information were somewhat better at predicting the passing decisions made in the second part of the survey than at predicting the decisions made in the first part of the survey. Because the players completing the second part of the survey were older and had been playing football for longer than those who dropped out, one might speculate that the passing decisions of more experienced players base upon perceptual information more heavily (or more consistently) than the passing decisions of less experienced players.

The illustrated approach can be adapted to analyzing data collected in real competition settings. Such data would overcome the restrictions that currently prevent the generalization of the reported estimates to real-world passing behavior. These restrictions relate to the top view used, the static game scenarios, and the lack of time restrictions on the passing decisions. However, the results suggest maintaining the hypotheses and testing them in studies using data with higher ecological validity. Such data could be collected via GPS or video-tracking systems during real-world competions. The position data of all players on the field at the moment of each pass and the information regarding who makes the pass to whom would be sufficient to compute all the perceptual variables used in this study. They could be computed with data processing software such as, for example, MATLAB (Mathworks).

The variables representing the perceptual information sources considered in the analyses are not exhaustive. One implication of research is the need to define further variables representing perceptual information that is potentially relevant to passing decisions. Example variables could relate to the team members' bodily or visual orientations, running directions, or speeds. Such variables could easily be included as additional predictors in regression models such as the one presented in this study. There is also a multitude of content representing internal information sources that would be interesting to test in future studies. Clearly, athletes possess more differentiated knowledge about their team members than that represented by the people model construct considered here. According to theory, the activation of this internal knowledge is dynamic and depends on the knowledge's relevance to the current game situation (Eccles and Tenenbaum, 2004). One implication for future studies is the need to address this dynamic and consider the multiple and more specific types of information that athletes have regarding the game and their team members. For example, knowledge about the specific strengths (e.g., speed, aerial power) of team members is likely influential, depending on these strengths' relevance in given game situations. The effects of specific types of knowledge when they relate to game contexts to differing degrees could be tested.

While the considered predictor variables significantly contributed to passing decisions, approximately 7% of the actual passing decisions were, based on the regression models' predictions, chosen from among the three lowest-ranked passing options within a given situation. This means that the predictor variables were not able to predict these passing decisions satisfactorily. It remains unclear what characterizes this 7%. Within a scenario-based study, it is not possible to determine whether a passing decision will result in a completed pass or whether it optimizes a team's situation by creating new opportunities or organizing the playmaking. This is not true of real game data. Using real game data, it will be possible to explore whether passes with deviating relationships to the predictor variables (as indicated by their low option ranking) can be characterized according to qualitative criteria (e.g., poor passes as opposed to creative and unexpected passes). It is also possible to analyze different categories of passes (e.g., based on expert ratings or the number of outplayed opponents) separately. If such a differential perspective reveals specific relationships between high-quality passes and certain perceptual information, then this indicates the need to deploy attentional strategies with a special focus on this perceptual information.

A final implication for future research is the need to extend the presented approach to other parameters that are relevant to team performance. The behavior of the athletes receiving the ball, the positioning of athletes involved in building defensive lines, and the running paths of forwards are potential dependent variables that could be analyzed by adopting the presented approach. Assuming that behavior emerges within an extended decisional process in interaction with environmental and knowledge-based information (Nitsch, 2004; Araújo et al., 2006; Johnson, 2006), further research studying the roles of the various information sources in various task-relevant decisions is needed.

To conclude, this article provides a research template with which to estimate the relevance of different information sources to passing decisions. The tested hypotheses of coexisting effects on the part of perceptual and knowledge-based information sources should be maintained and adapted for tests using data with higher ecological validity. The weighting and integration of external and internal information for passing decisions (and, of course, other decisions) remains largely unknown and is thus an open research question. In this sense, the present work is a preliminary step toward testing Steiner et al.'s (2017) integrative perspective empirically and eventually transforming it into an empirically grounded theory that considers the role of environmental information sources and athletes' associative knowledge on decision making in sports.

## Author contributions

SS conceptualized and designed the study. SS drafted the work and provided approval for the version to be published. SS agrees to be accountable for all aspects of the work in ensuring that questions related to the accuracy or integrity of any part of the work are appropriately investigated and resolved.

### Conflict of interest statement

The author declares that the research was conducted in the absence of any commercial or financial relationships that could be construed as a potential conflict of interest.

## Footnotes

^1^The assumption for the first variable is that passes are straight balls played along the ground.

^2^Each time the distance to the ball carrier changes into the next quintile, the odds of receiving a pass are multiplied by 0.726. A maximum four-unit-increase in distance to the ball carrier would be associated with 0.726^4^ = 0.278 times the odds of receiving a pass (Cohen et al., 2003). The reciprocal of 0.278 (1/0.278) indicates the change in odds in the opposite direction (e.g., a four-unit decrease from the most distant team members to those closest to the ball carrier).
